# Over-The-Counter (OTC) Drug Consumption among Adults Living in Germany: Results from the German Health Interview and Examination Survey for Adults 2008–2011 (DEGS1)

**DOI:** 10.3390/pharmacy6020052

**Published:** 2018-06-07

**Authors:** Eva Barrenberg, Hildtraud Knopf, Edeltraut Garbe

**Affiliations:** 1 Department of Clinical Epidemiology, Leibniz Institute for Prevention Research and Epidemiology—BIPS, Achterstraße 30, 28359 Bremen, Germany; barrenberg@leibniz-bips.de; 2 Department of Epidemiology and Health Monitoring, Robert Koch-Institute, General-Pape-Straße 62-66, 12101 Berlin, Germany; knopfh@rki.de; 3 Core Scientific Area ‘Health Sciences’ at the University of Bremen, Grazer Str. 2, 28334 Bremen, Germany

**Keywords:** non-prescription medicines, OTC drugs, self-medication, drug utilization studies, Germany, DEGS1, epidemiology, pharmaceutical public health

## Abstract

In order to assess the effects of prescription-only (Rx) to over-the-counter (OTC) drug switches and related policies, it is imperative to distinguish self-medication from OTC drug use. The objective of this study was to estimate the OTC drug use in the adult population in Germany, to identify its predictors and to highlight methodological differences when compared to the study of a self-medication prevalence. Seven-day prevalence of OTC drug use was calculated on the basis of information provided by 7091 participants of the German Health Interview and Examination Survey for Adults (DEGS1) conducted between 2008 to 2011. Logistic regression analysis was used to identify predictors of OTC drug use. Seven-day prevalence of OTC drug use was higher in women (47.16%) than in men (33.17%). Female gender, an age of more than 60 years, reduced health status, Rx drug use, and multi-morbidity were identified as predictors of OTC drug use. The levels of OTC drug use were higher than the self-medication prevalence found in the same data set probably because some OTC drugs are commonly prescribed by physicians. Drug utilization studies should, therefore, make a methodological distinction between self-medication and OTC drug use depending on whether the focus is on drug safety or the impact of regulatory decisions on the trade status.

## 1. Introduction

The use of over-the-counter (OTC) drugs is a subject of high relevance to public health policy. Over past decades, many active ingredients, which previously had been prescription-only (Rx) drugs, have become available OTC [1,2,3]. At the same time, European health care systems have revised their policies on the reimbursement of OTC drugs [4]. In order to monitor the effects of these policy changes in the general population, it is important to assess the prevalence of OTC drug use and possible predictors thereof.

Apart from a few exceptions, OTC drugs have not been reimbursable by statutory health insurance funds in persons over the age of 12 years in Germany since 2004 [5]. In adults and children over the age of 12 years, the use of OTC drugs can, therefore, not be measured with routinely collected data from statutory health insurance funds. In order to trace the effects of Rx-to-OTC switches and related policies, reimbursement decisions and health policies linked to OTC drugs, it is imperative to distinguish self-medication from OTC drug use and to monitor both types of medication use separately. Despite that fact, all population health surveys representative of the adult population living in Germany have focused on self-medication rather than OTC drug use so far. For instance, the MONICA study of 1990 and the German National Health Interview and Examination Survey of 1998 (GNHIES98) provided seven days of prevalence data about self-medication of 13.1% among 30-year-olds to 64-year-olds and of 34.6% among adults aged 18 to 79 years, respectively [6,7]. In 2013, data on medication use ascertained in the first wave (2008–2011) of the German Health Interview and Examination Survey for Adults (DEGS1) were published [8]. In this study, 58.8% of the population reported use of ‘prescribed preparations’ and 38.8% use of ‘self-medication’ in the previous seven days [8]. ‘Prescribed preparations’ were defined as those prescribed by medical doctors or naturopaths as well as previously prescribed products from family medicine cabinets. ‘Self-medication’ comprised preparations that were self-purchased without a medical prescription in pharmacies, supermarkets, or drugstores as well as drugs from family medicine cabinets that had not been previously prescribed. Differently from the definition of OTC drugs used in our research, self-medication in that study also included “dietary supplements such as vitamins or minerals” [8], medicinal products, and functional foods [8]. The assessment of self-medication in the DEGS1 study and its predecessors relied on information from study participants on how they had obtained the product in question rather than on the official trade status such as Rx status, OTC status, or differentiation from products not considered drugs. 

In contrast, little effort has been made to ascertain OTC drug use among German adults. In a 2008 telephone health survey in North Rhine-Westphalia, 38.7% of participants aged 18 years and older reported having used OTC drugs in the previous four weeks [9]. This survey was representative but limited to the population of only one Bundesland (Federal State). A recent attempt to study OTC drug use rather than the self-reported origin of the products was an online-survey carried out in 2013 among 300 German adults, which found a 7-day OTC drug use prevalence of 46.3%. While the study included participants from all over Germany, it cannot be considered representative due to the small sample size and sampling issues inherent to online surveys [10]. 

The present research study seeks to complement the results of the 2008–2011 DEGS1 [8] and of the online survey from 2013 [10] by analyzing OTC drug use instead of self-medication within the DEGS1 data set. It aimed to provide estimates of the prevalence of OTC drug use within a representative sample of German adults as well as to identify factors influencing their OTC drug use. 

## 2. Methods

DEGS1, which was conducted by the German Robert Koch Institute between 2008 and 2011, used a two-stage sampling method with examination centers across Germany with the goal of achieving high representativeness of the German population between 18 and 79 years. It consists of standardized, computer-assisted personal interviews (CAPI) on self-reported medical diagnoses and on medicine use, self-administered questionnaires about demographics, self-assessed health, health-related behavior and well-being, life situation, clinical exams, and laboratory analyses. The study design and sampling method of DEGS1 have been described in depth elsewhere [11,12,13,14]. 

In the invitation letters, the study participants were informed about the objectives and the examination procedures. The study participants received a document about data protection at the examination centers. If they had any questions, they could ask the examination teams at the Robert Koch-Institute or the staff in the examination centers. Participants provided written informed consent prior to the interview and examination.

**Figure 1:**
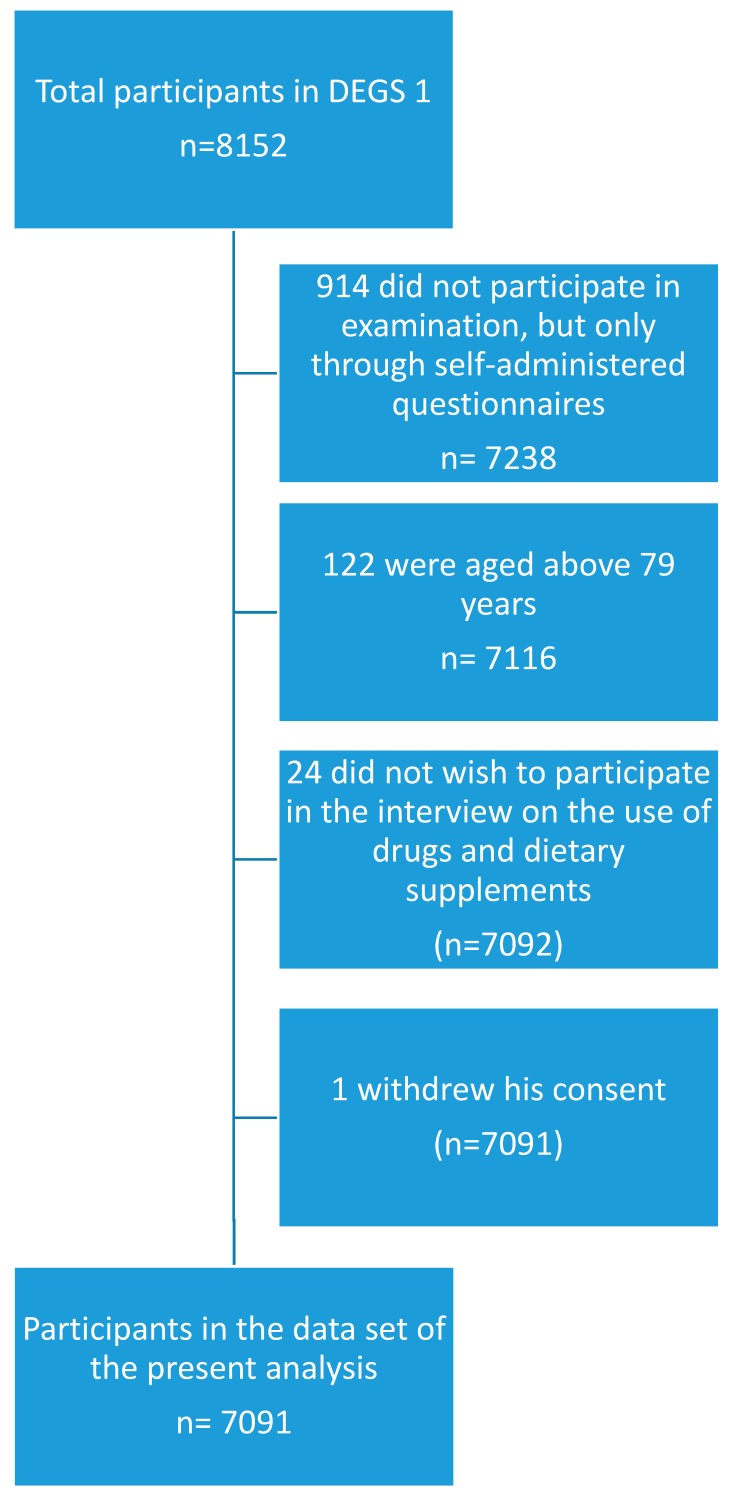
Flowchart of inclusion and exclusion of study participants.

The present study was based on a sub-sample of the DEGS1 study population, which additionally participated in a standardized CAPI on the use of drugs and dietary supplements [8]. Participants were invited to bring the original packages of all health-related preparations used in the previous seven days and were asked the following question during the CAPI: “Did you use medicines or dietary supplements such as vitamins or minerals during the last seven days? Please also think of painkillers, insulin-containing preparations, injections, and plant-based products. Please also mention preparations from supermarkets or drug stores.” The question was repeated until the participants did not mention any further products. The registration of drugs and other products in the drug use survey was based on the drug registration database AmEDa (Arzneimittel Erfassungs-Datenbank), which is a software product developed by the Robert Koch-Institute drawing on a set of drug master files made available by the Wissenschaftliches Institut der AOK (Scientific institute of the umbrella organization of several German statutory health insurance funds, WIdO) and on the database on dietary supplements of the German National Food Consumption Survey of the MRI, which is the German Federal Research Institute of Nutrition and Food (Max-Rubner Institut). If a product could not be identified with certainty at the study site, a follow-up via mail or telephone was conducted to complete missing information [8]. All persons aged between 18 and 79 years with valid data on the use of drugs and dietary supplements in the last seven days before examination were included in the sub-sample used for the present analysis. Persons who did not take part in the interview on drug usage were excluded. A flowchart on inclusion and exclusion of participants is presented in Figure 1. 

For each product, the interviewers scanned the German identification number for pharmaceutical products (Pharmazentralnummer, PZN). Drawing on AmEDa, the software retrieved information on the name, the pharmaceutical company, the pack size, and the pharmaceutical form. Where no PZN was available or information was lacking in the databases provided by WIdO or MRI, the interviewers filled-in the data manually into AmEDa. Further details regarding the design and method of the DEGS1 drug use survey can be found in the following references [8,15]. On the basis of this information, this study differentiated between prescription drugs, OTC drugs, and products were not considered drugs. As reference for the classification of trade status, annex 1 of the Ordinance on Prescription-Only Medicines (Arzneimittelverschreibungsverordnung, AmVV), which lists all substances subject to Rx status and possible exceptions thereto, was used as the primary source of information. In addition, the drug search engine of the AOK [16], the “Handbuch Rezeptfreie Medikamente” (Handbook of OTC drugs) [5], “Scribas Tabelle” (a German database of Rx drugs) [17], summaries of product characteristics [18], information from manufacturers and vendors, information from online pharmacies and drug stores, and pay-for-access information on vitamin and mineral products compiled by a German foundation for testing/consumer protection (Stiftung Warentest) were used. 

For the purpose of this paper, the definition of OTC drugs included both pharmacy-only drugs as well as general sales drugs including products classified as medicines that are available for sale in supermarkets and drug stores. Since these classifications may have been subject to change after and during data collection, coding, and analysis, the legal status as of 15 May, 2016 was considered valid for the products in question. Licensed homeopathic preparations were counted as drugs. Ophthalmologic products were either classified as Rx or OTC drugs. If study participants mentioned active ingredients/drug names/brands that could be either Rx or OTC drugs, the drugs were classified according to how participants reported having obtained them. If study participants mentioned active ingredients/product names/brands that could be either OTC drugs or dietary supplements, these entries were randomly assigned to either one or the other, according to the share of OTC drugs and dietary supplements of that specific active ingredient/drug name/brand on the German market. The latter information was obtained from pertinent pharmaceutical companies or vendors. If participants mentioned active ingredients that could be Rx drugs, OTC drugs, or dietary supplements and did not provide further specifications such as brand name or pharmaceutical form, such entries were classified as missing variables even if participants mentioned how they had obtained the product. 

As possible predictors for OTC drug use, education, net household income, migration status, type of health insurance, urbanization, socio-economic status, self-reported health, multi-morbidity, mental health disorders, alcohol consumption, and Rx drug use in the previous seven days were considered. The selection of possible predictors was partly based on finding from other studies on factors correlated with or predictors of OTC drug use and self-medication and partly on the authors’ own hypotheses. Information on education, household income, migration status, type of health insurance, and self-reported health was obtained from questionnaires filled in by the participants themselves. The reported highest level of education was classified based on the International Standard Classification of Education 1997 as defined by UNESCO and assigned to the categories “low,” “medium,” and “high,” according to the German micro-census categories [19,20]. For the level of urbanization, a distinction was made between “rural” (<5000 inhabitants), “small town” (5000–<20,000 inhabitants), “medium-sized town” (20,000–<100,000 inhabitants), and “city” (≥100,000 inhabitants). The classification of socio-economic status (SES) was based on education, profession, and household income [21]. The questionnaire also enquired whether participants or their parents were born abroad. On that basis, migration status was determined according to three categories classified as “none,” “one parent,” and “both parents.” People who were born abroad counted towards the latter category [22]. In the same questionnaire, participants were asked to provide information about their health insurance arrangements. This information was then classified into the three categories “statutory health insurance,” “private health insurance or health care scheme for civil servants (Beihilfe),” and “other” by the DEGS study team. Information on self-reported health statuses was based on the question “How is your health status in general?” with the answering options “very good,” “good,” “moderate,” “poor,” and “very poor.” 

In order to study multi-morbidity as a possible predictor of OTC drug use, information on health conditions and diseases was obtained through a physician-administered CAPI. For the purpose of our study, ‘multimorbidity’ [23,24] was defined as the presence of two or more of the following conditions: *in the last twelve months presence of* self-reported physician-diagnosed hypertension, coronary heart disease, cardiac insufficiency, diabetes, dyslipidaemia, circulatory disorders of the legs, asthma, thyroid disease, gastroduodenal ulcer, hepatitis, chronic inflammatory bowel disease, gout, rheumatoid arthritis, migraine, epilepsy, hay fever/allergic rhinitis, and neurodermatitis; *current medical care for* cancer, *ever diagnosed with* chronic renal insufficiency, arthrosis/degenerative joint disorders, osteoporosis, or Parkinson’s disease. A variable on mental health disorders was based on participants’ reports of the presence of one or more of the following physician-diagnosed illnesses: eating disorders, anxiety disorders, depression, and burn-out syndrome. The alcohol consumption was assessed using the AUDIT-C methodology and the results were classified into the categories “never,” “moderate,” and “risk behavior” [25]. 

To account for differences in terms of age, gender, region, citizenship, urbanization, and education between the study sample and the general German population between 18 and 79 years (as of 31.12.2010) as well as for the two-staged sampling approach, which is a weighting factor introduced for the DEGS1 study [15]. Percentages and means were calculated taking this weighting factor into account using the survey procedure of Stata. Possible predictors of OTC drug use were studied through logistic regression analysis using a manual forward approach. The order of possible independent variables to be included in the logistic regression model was based on results from unadjusted Student t-tests and chi-square tests at the sample level including those variables with the highest significance level first. Variables with equally low *p*-values of *p* < 0.000 found in the unadjusted tests for inference were included in the following order: gender, age, urbanization, self-reported health, multi-morbidity, and mental health disorders. 

For the logistic regression analysis, the above-mentioned weighting factor was applied throughout by using the Stata survey procedure to ensure the results are valid at the population level. To assess goodness-of-fit of the respective models at each stage of model building, the Archer and Lemeshow *F*-adjusted mean residual test for binary logistic regression models fitted to survey data [26,27] was used through the post-estimation *estat gof* command of Stata. An increase in goodness-of-fit was used as an inclusion criterion for the variables. For the Archer and Lemeshow *F*-adjusted mean residual test, a *p*-value of <0.05 indicates a lack of fit. For means and odds ratios, a *p*-value of <0.05 or a lack of overlap of 95% confidence intervals were considered indicative of statistical significance. All statistical analyses were conducted with Stata 13. 

## 3. Results

### 3.1. Study Population

Out of 7116 participants aged 18 to 79 years who took part in the physical examination of DEGS1, 7091 also participated in the interview on the use of drugs and dietary supplements. Their demographic characteristics are summarized in Table 1.

### 3.2. Drug Use Prevalence

The 7-day prevalence of having used at least one OTC drug was 40.2% in total and 33.2% and 47.2% for men and women, respectively. For the use of at least one Rx drug, the seven-day prevalence was 45.4% among men, 69.7% among women, and 57.6% among the total population. Regarding the dietary supplements, their use in the previous seven days was found to be 11.0% of men, 20.4% of women, and in 15.7% of the total population. The mean numbers of OTC drugs used in the past seven days in both genders was 0.67 (95% CI: 0.63–0.71), 0.47 (95% CI: 0.43–0.50) in men, and 0.87 (95% CI: 0.81–0.93) in women. The gender difference was statistically significant. An overview of the seven-day prevalence per number of drugs used is provided in Table 2. 

### 3.3. Factors Associated with OTC Drug Use

All variables presented in Table 1 were considered for inclusion in a logistic regression model. The best-fit model found the following statistically significant predictors for the use of at least one OTC drug in the previous seven days: female gender, multimorbidity, good, moderate, poor, and very poor self-reported health in comparison to very good self-reported health, ages 60–69, and 70–79 years, and Rx drug used in the previous seven days (see Table 3). 

The *F*-adjusted mean residual test statistic of the presented model was 0.25 (*p* = 0.9869). Urbanization, alcohol consumption, and mental health disorders were not included in the final model since these variables lowered the goodness-of-fit. Household income was included in the model rather than education status or socio-economic status since it made, in comparison, the greatest contribution to goodness-of-fit. 

## 4. Discussion

Our study examined for the first time OTC drug use in a representative sample of the adult population living in Germany. We found a seven-day OTC drug use prevalence of 40.2%. Female gender, older age, reduced self-reported health status, and multi-morbidity were significant predictors of OTC drug use among that population.

The finding of a seven-day OTC drug use prevalence of 40.2% was unexpected, as Knopf et al. had, based on the same data set and the broader definition of ‘self-medication’ instead of ‘OTC drug use’, only found a seven-day self-medication prevalence of 38.8% [8]. In order to better understand these findings, the differences in the methods of the two studies require a closer look. In both cases, data were collected by asking participants whether they had used medicines or other health-related products during the last seven days including preparations from supermarkets or drug stores. For the purpose of our study, additional information on PZN, name, pharmaceutical company, pack size, and pharmaceutical form was then used to determine the trade status of the respective product, which is defined as whether the product was an Rx drug, an OTC drug, or no drug at all. Knopf et al., in turn, did not classify the drugs into Rx drugs and self-medication according to their trade status but relied on information by study participants on how they had obtained the products. For this classification, participants could specify one of the following options: (i) prescribed by a physician; (ii) recommended by a naturopath; (iii) bought without a prescription, (iv) from the family medicine cabinet previously bought without a prescription; (v) from the family medicine cabinet previously prescribed; (vi) other; and (vii) unknown. When analyzing the seven-day prevalence of self-medication, Knopf et al., therefore, summarized categories (iii) and (iv) into the category of self-medication regardless of the actual trade status of the products mentioned in these categories. The assumption that self-medication as such includes a larger variety of products than OTC drug use since it also includes dietary supplements and other product falls, however, short of the fact that there may be drugs and homeopathic products that are generally prescribed or recommended by physicians or naturopaths, they have OTC status. We believe that these methodological differences in classifying OTC or self-medication use explain the discrepant and unexpected results based on the same data set.

For the purpose of this study, homeopathic preparations were classified as either Rx or OTC drugs depending on the dose (homeopathic potentisation), according to annex 1 of the AmVV. However, homeopathic preparations were not counted towards self-medication by Knopf et al. if they had been recommended by naturopaths. Among the DEGS1 study population, 1.6% used products recommended to them by naturopaths. In the majority of cases, these are OTC products since naturopaths cannot prescribe Rx products unless they are also licensed physicians. 

The discrepant results between this study and the study by Knopf et al. could also be due to drugs that are OTC in terms of their trade status but are, nonetheless, commonly prescribed by physicians. In the DEGS1 study population, 16.7% of participants had used at least one OTC drug in the previous seven days that had been prescribed or recommended by a physician. For example, it is likely that low-dose acetylsalicic acid (ASA) was often “prescribed by a physician” and, therefore, did not qualify for the classification of “self-medication,” according to the categories defined by Knopf et al. However, low-dose ASA is an OTC drug, according to its trade status. ASA in a dose of up to 300 mg can be reimbursed by statutory health insurance funds in patients with stable coronary heart disease or following myocardial infarction or stroke despite its OTC status [5,28] resulting in higher physician prescribing of this OTC drug. Despite the generally low price per pack, which can occasionally be below the supplementary payment for prescription drugs, a significant amount of low-dose ASA is still prescribed. According to the German Drug Prescription Report (Arzneiverordnungs-Report) from 2012, 618.8 million defined daily doses of low-dose ASA were prescribed and charged to statutory health insurance funds in 2011, which is the last year of the data collection phase of the DEGS1 study [29].

Despite the seemingly little difference between the self-medication and OTC drug use prevalence and the possible explanations for this finding presented above, a 40.2% OTC drug use prevalence presents an important result in German pharmacoepidemiologic research and has implications for the greater public health context. For example, because only due to the methodological distinction, we were able to show that OTC drug use concerns a significant part of the population while it would also have been conceivable to find a much lower prevalence of OTC drugs used compared to that of self-medication, which may actually be the case in other countries. However, considering that the policy is made on the basis of different trade statuses rather relying on a distinction of self-medication from non-self-medication and that OTC drug use in the population is significant, our work supports the argument that OTC drug use should receive more attention in public health policy, which is currently rather limited. 

The analysis of the number of products used suggests that polypharmacy with OTC drugs does not seem to present a major concern since the majority of OTC drug users limit their consumption to one product within a seven-day time frame. 

OTC drug use was significantly higher among people aged 60 years and older. Moreover, multi-morbidity was identified as a significant predictor for OTC drug use. While most diseases and conditions included in our definition of multi-morbidity require treatment with prescription drugs, there are also OTC options for some of them in Germany. For example, there are OTC drugs for the treatment of coronary heart disease, circulatory leg disorders, gastroduodenal ulcers, and hay fever/allergic rhinitis such as low dose ASA, proton pump inhibitors, or second-generation antihistamines [5]. Both older people and those affected by multi-morbidity may represent vulnerable population groups with regard to OTC drug use, which is seen by contraindications or precautions and warnings that refer to certain age groups or conditions and diseases, which can be found in the summary of product characteristics of several OTC drugs [3]. Therefore, the finding of our study that multimorbidity and higher age are predictors of OTC drug use is of high relevance to regulators making decisions on Rx-to-OTC switches. To date, there is no specific guidance on Rx-to-OTC switches in Germany. However, acknowledging the significant share of OTC drug users in the population, it would be advisable that such policies were developed in the form of criteria guiding the German Expert Advisory Committee on Prescription-Only Issues in its deliberations when advising on switch applications. Such criteria should then also include considerations regarding a higher age and multi-morbidity to ensure that risks from OTC drug use for the population will be adequately assessed.

OTC drug use was also significantly higher in any self-reported health category that was worse than “very good.” This finding contradicts that of a Spanish study, which found significantly higher levels of self-medicated analgesic use among adults with good or excellent health status [30]. However, it is well-known that the measure of self-reported health is subject to important cultural influences and unadjusted cross-country comparisons are, therefore, difficult to make [31].

Drug use was generally significantly higher among women with regard to OTC drugs, Rx drugs, and dietary supplements, which are in line with previous findings from Germany and elsewhere [4,6,7,8,9]. Income, education, and SES were studied separately as possible predictors for OTC drug use because SES is a variable composed of education, income, and professional status. We did not identify income as a significant predictor for OTC drug use but the variable made a more powerful contribution to the goodness-of-fit of the logistic regression model than the other variables. This finding is quite important since an association between higher SES and self-medication has been reported in studies from Germany before [7,9] while no association between education and OTC drug use has been found [10]. It is conceivable that there is an association between income and OTC drug use for which the finding of the OR of 1.26 (95% CI: 1.06–1.51, *p* = 0.01) for a household income of <2500 ≤3500 Euros, which may also be considered indicative, but that it could not be shown in our model due to the application of the linearizing weighting factor. It would be desirable to better understand the respective influence of professional status, education, and income on OTC drug use and their implications for public health policy and practice with regard to safe use, compliance, oversupply, and equitable access. For instance, it may be the case that people who are able to afford OTC drugs are using more than necessary while others are unable to afford an OTC drug despite a medical need.

Similar to the association with income, education, and SES, urbanization as a proxy for easier access to OTC drugs did not add to the goodness-of-fit of the logistic regression model, but it may be worthwhile to study other possible proxy measures for accessibility such as purchasing OTC drugs over the internet and whether such purchases are related to higher OTC drug use as well as whether easier accessibility of OTC drugs affects their safe use. Rx drug use in the previous seven days was identified as a significant predictor of OTC drug use. This finding is in line with that of an online survey on OTC drug use in Germany [10]. A statistically significant association between OTC drug use and alcohol consumption was found at the sample level, but, when included into the logistical regression model, alcohol consumption neither added to the goodness-of-fit of the model nor were the odds ratios statistically significant after applying the linearizing weighting factor. The variable was therefore not included into the final logistic regression model. The question of whether there is an association between OTC drug use and alcohol consumption may nonetheless merit further attention in future studies, but it is unclear whether such an association had any implications for clinical or regulatory practice. In a review article by Prescott, for instance, it was shown that there is no evidence of acute hepatotoxic incidents following paracetamol use at therapeutic dosages in alcoholics [32].

As with previous population-based German studies on drug use [6,7,33,34], the DEGS1 survey was intended to study self-medication and prescribed medication. While it is often desirable to use harmonized methodologies in drug utilization studies to enable comparisons across different times and locations, using different methodological approaches may provide new perspectives by emphasizing other aspects of drug use. The approach of studying the use of self-medication versus prescribed/recommended medication based on prescription or recommendation vs. self-purchase as pursued by Knopf et al. is particularly useful when questions of OTC drug safety are of concern. In such cases, the question of whether patients received instructions from their physicians rather than the trade status of the drug of concern will play an important role. The classification approach of OTC and Rx drugs based on their trade status, in turn, can inform or support the evaluation of changes to drug law since changes to trade status are made along the lines of legal distinctions between Rx status, OTC status, and their delineation from non-drugs. Considering the complexities of the OTC drug market and the different products and therapeutical categories, it is obvious that an evaluation of an actual Rx-to-OTC switch or another substance-specific regulatory decision would require studying the epidemiology of that particular drug or group of drugs. However, the analysis of OTC drug use as seen in the present study can provide guidance for the regulation of the legal category of OTC drugs such as deciding what considerations need to be made for assessing applications to changing drug trade statuses. 

Regularly conducted population health surveys present a unique opportunity for monitoring OTC drug use, which cannot be measured by routine data from statutory health insurance funds. Moreover, the DEGS1 data set also includes –to a limited extent– information on medication not being part of the formal German drug market. However, it was beyond the scope and the technical and financial possibilities of this study to estimate the prevalence and predictors of inappropriate drug use. However, it would be desirable to focus on these topics in future research in the field of OTC drugs. 

While the specific usage patterns and regulations concerning OTC drugs are unique to each country, this study adds value to international pharmaceutical public health through the general methodological insight that self-medication and OTC drug use are not equivalent concepts and that, empirically, a corresponding population prevalence may differ. 

### Strengths and Limitations

The present study has several strengths such as relying on the elaborate design and the large sample size of the DEGS1 survey, which is representative of the adult population in Germany. A limitation, however, is the upper age limit of 79 years in the study population. Since we could show a strong correlation between OTC drug use and higher age, it is likely that the true effect has been underestimated in this study. Similarly, we identified multi-morbidity as an important predictor for OTC drug use but the true effect may be larger than reported here since patients with multi-morbidity may be affected by reduced mobility and, therefore, may have been unable to travel to DEGS study sites. However, the high level of the goodness-of-fit of the logistic regression model shows that, overall, the predictors of OTC drug use have been selected appropriately. 

Another positive feature of the DEGS1 survey is that information about drugs used in the previous seven days was collected in CAPI interviews. Furthermore, participants were invited to bring the packages of the drugs used to the interview where they could be scanned, which allows for semi-automatized collection of data. By scanning the packages, the relevant information including was provided for 73% of the preparations while 27% had to be subsequently researched and manually entered [8]. A limitation is that the DEGS1 survey questionnaire contained a filter variable asking whether a drug was used regularly or occasionally. During CAPI interviews of DEGS1, further questions about the actual use of drugs were only asked if they were used regularly [35]. That, however, is not a common feature of OTC drug use [3,36] and, thereby, prevented a more in-depth analysis of OTC drug use.

Despite a greater completeness of the medication data collected at the study sites, telephone and mail follow-up was conducted for 1.5% of the participants [8] to ensure even greater completeness of the information on drugs used, which presents a strength of this study. However, whether a product used had Rx or OTC status was not directly recorded. Nevertheless, in the context of our study, the trade status of each product was carefully assessed on the basis of various information sources. This permitted the differentiation of OTC and Rx drug use and the subsequent analysis of OTC drug use which previously could not be done.

## 5. Conclusions

Drug utilization studies should not consider ‘self-medication’ and ‘OTC drug use’ as synonyms. Rather, both concepts should be monitored separately depending on the aim of the health policies they seek to inform. For example, the question of how patients obtained certain drugs is different from how legal provisions on trade status translate into drug consumption patterns in practice. While the former question can improve the evidence base for questions of drug safety where the interaction with a physician is pertinent, the latter can help assess the impact of regulatory decisions on trade status. Distinguishing self-medication and OTC drug use in research of public health practice is, therefore, not trivial. Moreover, significant amounts of OTC drugs may actually be prescribed or recommended by physicians, which merits further scientific attention.

With a 40.2% prevalence of OTC drug use, the latter makes a substantial contribution to the pharmaceutical supply in Germany and the safe use of OTC drugs should, therefore, receive the attention in public health research and sanitary consumer protection it deserves. However, OTC drug use is rarely the focus of drug utilization studies. It should be also considered that opportunities to study OTC drug use are more limited than those utilized for studying Rx drug use. In order to enhance the scarce empirical evidence on OTC drug use in Germany with future population-based studies, it would be valuable to amend the drug use survey questionnaire by including the legal status of drugs and by collecting further information on drugs used only occasionally. Collecting both types of information would permit specific analyses depending on the health policy question they seek to inform.

With odds ratios of 1.6 and above, female gender, an age of 60 years and older, a moderate, poor, and very poor health status were the five strongest predictors for OTC drug use. These findings can be useful in developing regulatory guidance on Rx-to-OTC switch decisions. In addition, the associations between older ages and multi-morbidity and OTC drug use should be considered in future policy decisions on OTC drugs in Germany.

## Figures and Tables

**Table 1:** Study population.

Gender	n	Weighted Percentage
Male	3399	49.7
Female	3692	50.3
Age groups		
18–29	1065	18.8
30–39	838	14.7
40–49	1293	21.5
50–59	1394	18.2
60–69	1393	14.1
70–79	1108	12.7
Urbanization		
Rural (<5000 inhabitants)	1301	15.3
Small town (5000–<20,000 inhabitants)	1711	24.0
Medium-sized town (20,000–<100,000 inhabitants)	2069	29.3
City (≥100,000 inhabitants)	2010	31.4
Self-reported health status		
Very good	909	14.2
Good	4300	60.5
Moderate	1646	22.7
Poor	173	2.3
Very poor	22	0.3
Migration status		
None	5854	80.1
One parent	311	4.8
Both parents	699	15.1
Socio-economic status		
Low	1129	19.6
Medium	4246	60.4
High	1672	19.9
Education		
Low	1003	21.1
Medium	3773	55.1
High	2271	23.7
Net household income (Euros)		
≤1500	2030	29.1
>1500 ≤2500	2388	33.2
>2500 ≤3500	1406	20.2
>3500 ≤4500	661	9.2
>4500 ≤5500	355	4.7
>5500	251	3.5
Type of health insurance		
Statutory health insurance	6055	87.7
Private health insurance or health care scheme for civil servants (Beihilfe)	841	11.6
Other	49	0.8
Multimorbidity		
Yes	2467	30.8
No	4624	69.2
Mental health disorders		
Yes	506	7.7
No	6585	92.3
Alcohol consumption		
Never	777	13.0
Moderate	3794	53.7
Risk behaviour	2252	33.4

**Table 2:** Seven-day prevalence of use of OTC drugs, Rx drugs, and dietary supplements per number of products used.

	Number of Products	OTC Drugs			Rx Drugs			Dietary Supplements		
		n	Prevalence (Weighted Percentage)	Weighted Mean (95% CI)	n	Prevalence (Weighted Percentage)	Weighted Mean (95% CI)	n	Prevalence (Weighted Percentage)	Weighted Mean (95% CI)
Men	0	2195	66.8	0.47 (0.43–0.50)	1643	54.5	1.18 (1.10–1.26)	2964	88.9	0.15 (0.13–0.18)
	1	853	24.1		560	16.6		326	8.5	
	2	226	5.9		374	9.8		68	1.5	
	3	87	2.4		281	7.1		23	0.4	
	4	21	0.5		232	5.4		10	0.4	
	≥5	17	0.3		309	6.6		8	0.1	
Women	0	1911	52.7	0.87 (0.81–0.93)	1045	30.1	1.66 (1.59–1.74)	2874	79.5	0.30 (0.27–0.33)
	1	1013	27.0		1044	29.6		558	14.1	
	2	427	11.7		635	16.9		174	4.5	
	3	191	4.8		367	9.1		57	1.3	
	4	62	1.5		245	5.7		18	0.4	
	≥5	88	2.2		356	8.5		11	0.1	
Total	0	4106	59.7	0.67 (0.63–0.71)	2688	42.2	1.42 (1.36–1.48)	5838	84.2	0.23 (0.21–0.25)
	1	1866	25.6		1604	23.1		884	11.3	
	2	653	8.8		1009	13.3		242	3.0	
	3	278	3.6		648	8.1		80	0.9	
	4	83	1.0		477	5.5		28	0.4	
	≥5	105	1.2		665	7.6		19	0.1	

**Table 3:** Factors associated with OTC drug use in the previous seven days among German adults aged 18–79 years.

Predictors for Using at Least One OTC Drug	OR	95% CI	*p*
Gender			
Male	Reference		
Female	1.64	1.44–1.86	<0.001
Age group (years)			
18–29	Reference		
30–39	1.10	0.86–1.40	0.438
40–49	1.17	0.94–1.45	0.154
50–59	1.11	0.89–1.38	0.346
60–69	1.59	1.27–1.98	<0.001
70–79	2.14	1.69–2.71	<0.001
Self-reported health			
Very good	Reference		
Good	1.50	1.21–1.86	<0.001
Moderate	2.14	1.69–2.75	<0.001
Poor	1.75	1.10–2.80	0.020
Very poor	4.16	1.03–16.84	0.046
Multimorbidity			
No	Reference		
Yes	1.52	1.29–1.79	<0.001
Household income(Euros)			
≤1500	Reference		
>1500 ≤2500	1.05	0.90–1.24	0.541
>2500 ≤3500	1.26	1.06–1.51	0.010
>3500 ≤4500	1.14	0.87–1.50	0.352
>4500 ≤5500	1.36	0.97–1.91	0.078
>5500	1.22	0.85–1.76	0.277
Migration status			
None	Reference		
One parent	0.80	0.57–1.11	0.173
Both parents	0.90	0.71–1.14	0.391
Rx drug use			
No Rx drug use in the previous seven days	Reference		
Rx drug use in the previous seven days	1.30	1.11–1.51	0.001
Constant: OR: 0.1882222 *p* < 0.001 Goodness-of-fit test: *F* (9171) = 0.25, *p* = 0.9869			
